# P-1472. Comparable Vaccine Effectiveness of Adjuvanted and High-Dose Influenza Vaccines in Preventing Test-Confirmed Influenza Outcomes, including Hospitalization, in Overall and High-Risk Older Adults: A Test-Negative Design Study During 2022-2023 and 2023-2024

**DOI:** 10.1093/ofid/ofaf695.1658

**Published:** 2026-01-11

**Authors:** Mahrukh Imran, Benjamin Chastek, Tim Bancroft, Noah Webb, Stephen I Pelton, Mendel Haag, Ian McGovern

**Affiliations:** CSL Seqirus, Toronto, ON, Canada; Optum, Eden Prairie, Minnesota; Optum, Eden Prairie, Minnesota; Optum, Eden Prairie, Minnesota; Boston Medical Center, Boston, Massachusetts; CSL Seqirus, Toronto, ON, Canada; CSL Seqirus, Toronto, ON, Canada

## Abstract

**Background:**

In 2022, the US Advisory Committee on Immunization Practices (ACIP) recommended adults ≥65 years receive adjuvanted or higher-dose influenza vaccines. Increased uptake following this recommendation enabled the estimation of season-specific relative vaccine effectiveness (rVE) using a test-negative design (TND). A 2022–23 season TND study found comparable effectiveness of adjuvanted (aQIV) and high-dose quadrivalent influenza vaccine (HD-QIV) against test-confirmed influenza. Expanding on prior evidence, this study evaluated the rVE of aQIV vs. HD-QIV in preventing test-confirmed influenza during 2023–24, including hospitalizations in overall and high-risk older adults in a pooled analysis with 2022–23.

**Figure 1.**
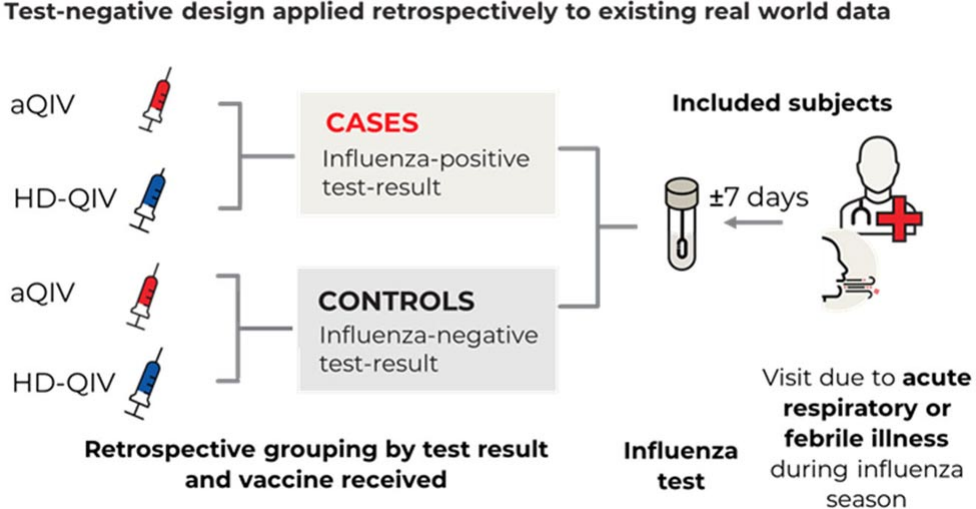
Study Design.

**Figure 2.**
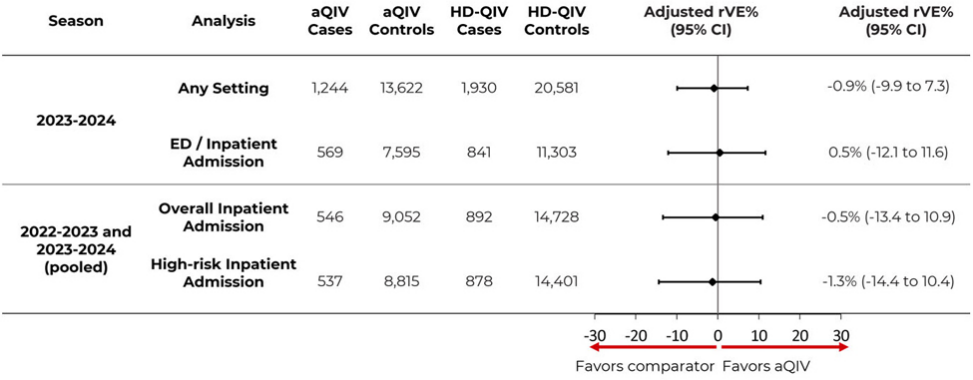
Adjusted rVE of aQIV vs. HD-QIV in preventing test-confirmed influenza.

**Methods:**

This retrospective TND study included US adults ≥65 years vaccinated with aQIV or HD-QIV who presented with acute respiratory or febrile illness in any setting or emergency department (ED)/inpatient settings (Figure 1). rVE was also evaluated in the inpatient only setting through a pooled analysis of 2022–23 and 2023–24 seasons. A subgroup pooled analysis additionally estimated rVE in inpatient settings in patients with ≥1 high-risk conditions. A doubly robust model was used, combining inverse probability of treatment weighting and logistic regression to adjust for potential confounders.

**Results:**

37,377 vaccinated patients were included (3,174 positive cases and 34,203 negative controls). Comparable effectiveness between aQIV and HD-QIV was observed in preventing test-confirmed influenza with a rVE of -0.9% (95% CI: [-9.9, 7.3]) in any setting and 0.5% (-12.1, 11.6) in ED/inpatient settings (Figure 2). Similarly, comparable effectiveness was observed in the pooled inpatient only analysis in both the overall (-0.5% [-13.4, 10.9]) and high-risk subgroup (-1.3% [-14.4, 10.4]) (Figure 2).

**Conclusion:**

Consistent with prior evidence, these results demonstrate comparable effectiveness between aQIV and HD-QIV for preventing test-confirmed influenza in any or ED/inpatient settings among adults ≥65 years during the 2023–24 season. Additionally, pooled analyses for the 2022-23 and 2023-24 seasons showed comparable effectiveness between aQIV and HD-QIV for hospitalizations, in overall and high-risk older adults.

**Disclosures:**

Mahrukh Imran, MScPH, CSL Seqirus: Grant/Research Support|CSL Seqirus: Stocks/Bonds (Private Company) Benjamin Chastek, MS, Optum (UnitedHealth Group): Stocks/Bonds (Public Company) Tim Bancroft, PhD, Optum: I am an employee of Optum. Optum was paid by Seqirus for this work. My employment at Optum is not contingent upon this work. Stephen I. Pelton, MD, CSL Seqirus: Advisor/Consultant|GSK: Grant/Research Support|GSK: Honoraria|Merck Vaccines: Grant/Research Support|Merck Vaccines: Honoraria|Pfizer, Inc.: Grant/Research Support|Pfizer, Inc.: Honoraria|Sanofi: Honoraria|Sanofi: DSMB, Adjudicator for RSV vaccine trial Mendel Haag, PhD, PharmD, CSL Seqirus: Grant/Research Support|CSL Seqirus: Stocks/Bonds (Private Company) Ian McGovern, MPH, CSL Seqirus: Grant/Research Support|CSL Seqirus: Stocks/Bonds (Private Company)

